# Genome sequences of *Arthrobacter globiformis* phages JuneStar and Pumpkins in Bismarck, ND

**DOI:** 10.17912/micropub.biology.001378

**Published:** 2024-11-12

**Authors:** Madeline Dojs, Christine Fleischacker, Celia Brekken, Ethan Emineth, Allison Hughes, Sarah Rodriguez-Brandon, Corrina Vigness

**Affiliations:** 1 Biology, University of Mary, Bismarck, North Dakota, United States

## Abstract

We report the isolation and characteristics of phages JuneStar and Pumpkins, siphoviruses isolated from soil in Bismarck, ND using *Arthrobacter globiformis* B2979-SEA. Based on gene content similarity, both phages are assigned to actinobacteriophage cluster AZ1. They encode a putative serine integrase that is conserved across cluster AZ1 phages, suggesting a temperate lifestyle.

**Figure 1. Particles of JuneStar and Pumpkins:**
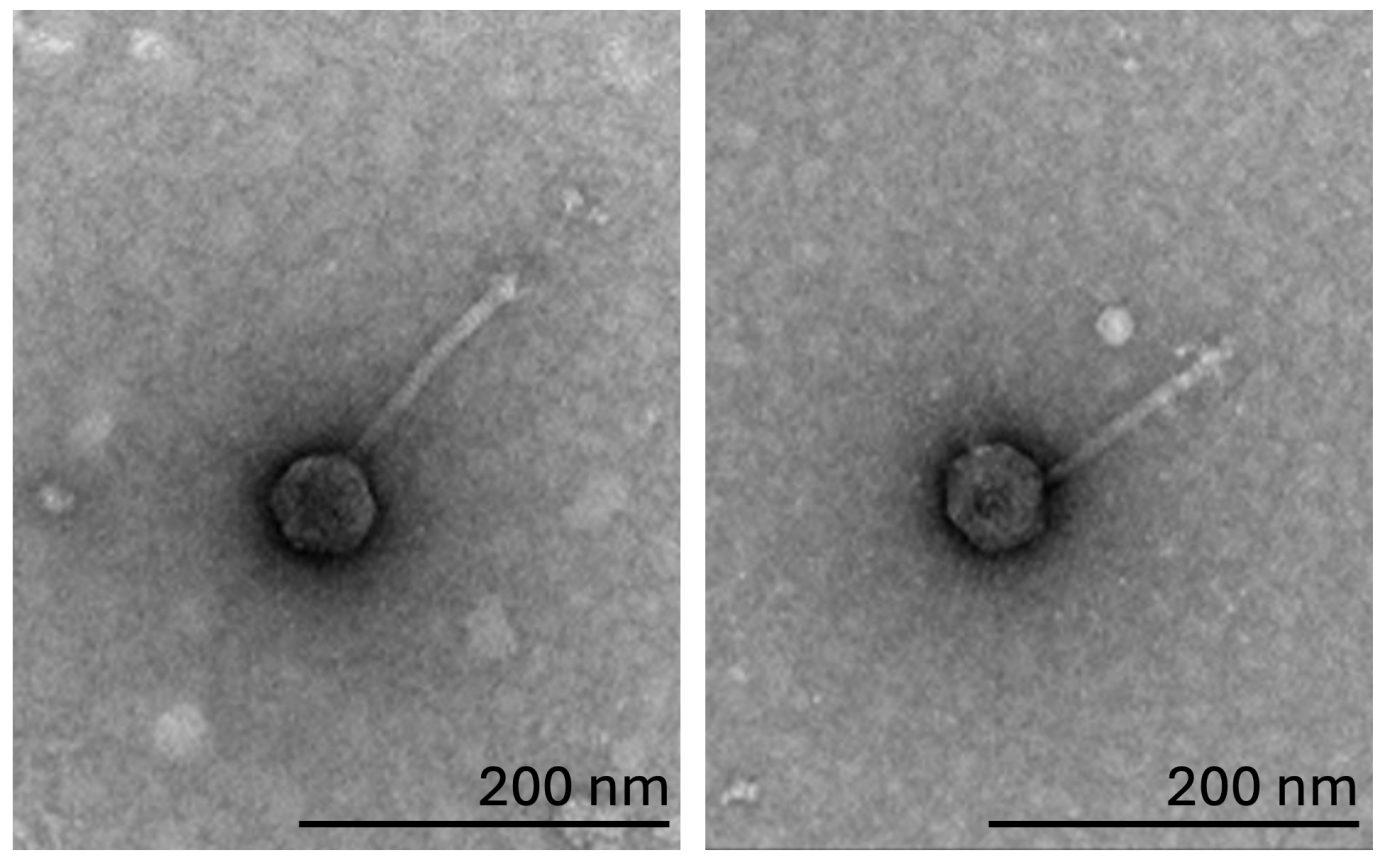
Negative-staining (1% uranyl acetate) transmission electron microscopy revealed Pumpkins (left) and JuneStar(right) to be siphoviruses. Pumpkins has a tail length of 131-138 nm and a capsid diameter of 53 - 59 nm (n=3) while JuneStar has a tail length of 122 nm and a capsid diameter 61 nm (n=1). Scalebar is 200 nm.

## Description

Bacteriophages (phages) are viruses that infect bacteria. Several phages that infect actinobacteria have been developed to treat antibiotic-resistant bacterial infections (Hatfull, 2022) . Here, we describe the isolation and characterization of two phages, JuneStar and Pumpkins, that infect *Arthrobacter globiformis* , an actinobacterium ubiquitous in soil and not known to cause disease in humans. (Jones, 2006)

Phages JuneStar and Pumpkins were isolated from soil samples collected on the grounds of the University of Mary in Bismarck, ND. JuneStar was extracted from fine, dark, and wet soil that was rich in decaying material (GPS 45.72573 N, 100.75322 W) whereas Pumpkins was extracted from moist soil with dead vegetation in a flower garden (GPS coordinates were 46.72378 N, 100.75231 W). Soil samples were washed with peptone-yeast extract-calcium (PYCa) liquid medium, and bacteriophages collected from the wash through a 0.22-µm filter. The filtrate was mixed with soft agar containing *Arthrobacter globiformis* B-2979, overlaid on PYCa agar, and incubated at 30°C for 48 hours (Poxleitner, 2018) . JuneStar produced 6mm clear plaques surrounded by a 1mm thick halo. While Pumpkins produced 13mm clear plaques with a 2mm thick halo. Phages were purified by three rounds of plating. Negative-staining (1% uranyl acetate) transmission electron microscopy showed a siphovirus morphology for both phages ( Figure 1 ).

Double-stranded DNA was isolated from lysates of JuneStar and Pumpkins using the Promega Wizard DNA Cleanup kit, prepared for sequencing using the NEB Ultra II Library Kit, and sequenced using an Illumina MiSeq (v3 Reagents), yielding 562,645 single-end 150-bp reads for JuneStar (1,895-fold coverage) and 782,025 single-end 150-bp reads for Pumpkins (2,667-fold coverage). Raw reads were assembled and checked for completeness using Newbler v2.9 and Consed v29, using default parameters (Russell, 2018) . Based on gene content similarity of at least 35% to phages in the Actinobacteriophage database (https://phagesdb.org/), both JuneStar and Pumpkins were assigned to the cluster AZ, subcluster AZ1 (Russell, 2017) (Pope, 2017) . The JuneStar genome is 44,517 bp long, with 11-base 3' overhang (CGAACCGGCAT) and G+C content of 68.3%, whereas the genome of Pumpkins is 43,976 bp with the same 11-base 3' overhang as JuneStar, and G+C content of 68.5%.

Genome annotation was performed with DNA Master (cobamide2.bio.pitt.edu) and PECAAN (discover.kbrinsgd.org), both of which utilize Glimmer-v3.02 (Delcher, 2007) and Genemark-v3.25 (Besemer and Borodovsky, 2005) to predict potential open-reading frames, as well as Phamerator (Cresawn, 2011) and Starterator (http://phages.wustl.edu/starterator/ref). ARAGORN v1.2.41 (Laslett, 2004) and tRNAscan-SE 2.0 (Lowe, 1997) were used to detect the presence of tRNAs. Gene functions were predicted with BLASTp (Altschul et al, 1990) searches against the Actinobacteriophage and NCBI non-redundant databases as well as HHPred v3.18 (Söding, 2005) searches against the PDB mmCIF70, Pfam-A, and NCBI Conserved Domain databases. All software was used with default parameters.

The genome of JuneStar contains 68 candidate protein-coding genes, 33 of which could be assigned functions, whereas the genome of Pumpkins contains 66 candidate protein-coding genes, of which 33 genes could be assigned functions. No tRNAs were identified in either genome. As with other cluster AZ1 phages, neither JuneStar nor Pumpkins encode an identifiable immunity repressor. However, based on the presence of a conserved serine integrase, experimentally validated lysogen-formation by cluster AZ1 phage Powerpuff (Kapinos, 2022) , and the presence of halos in the plaques, these phages are presumed to be temperate. A putative endolysin that is well-conserved across cluster AZ1 is either encoded across the first or second halves of the genomes. In JuneStar, this endolysin is similar to phage Elizi and is found within the first half of the genome (JuneStar 24), whereas in Pumpkins genome the endolysin is similar to phage PowerPuff and it is encoded within the second half of the genome (Pumpkins 56).

**Nucleotide sequence accession numbers**

The GenBank and Sequence Read Archive (SRA) accession numbers for JuneStar are PP978778 and SRX23452933, respectively. The GenBank and Sequence Read Archive (SRA) accession numbers for Pumpkins is PP978775 and SRX23452936, respectively.
